# Osmotic pressure induces unexpected relaxation of contractile 3D microtissue

**DOI:** 10.1140/epje/s10189-025-00497-0

**Published:** 2025-06-24

**Authors:** Giovanni Cappello, Fanny Wodrascka, Genesis Marquez-Vivas, Amr Eid Radwan, Parvathy Anoop, Pietro Mascheroni, Jonathan Fouchard, Ben Fabry, Davide Ambrosi, Pierre Recho, Simon de Beco, Martial Balland, Thomas Boudou

**Affiliations:** 1 Laboratoire Interdisciplinaire de Physique (LIPhy), Université Grenoble Alpes, CNRS, 38000 Grenoble, France; 2 Université Paris Cité, CNRS, Institut Jacques Monod, 75013 Paris, France; 3 Institut de Biologie Paris-Seine (IBPS), Sorbonne Université, CNRS, 75005 Paris, France; 4 Department of Physics, University of Erlangen-Nürnberg, Erlangen, Germany; 5 Dipartimento Di Scienze Matematiche, Politecnico Di Torino, Turin, Italy

## Abstract

Cell contraction and proliferation, matrix secretion and external mechanical forces induce compression during embryogenesis and tumor growth, which in turn regulate cell proliferation, metabolism or differentiation. How compression affects tissue contractility, a hallmark of tissue function, is however unknown. Here we apply osmotic compression to microtissues of either mouse colon adenocarcinoma CT26 cells, mouse NIH 3T3 fibroblasts, or human primary colon cancer-associated fibroblasts. Microtissues are anchored to flexible pillars that serve as force transducers. We observe that low-amplitude osmotic compression induces a rapid relaxation of tissue contractility, primed by the deformation of the extracellular matrix. Furthermore, we show that this compression-induced relaxation is independent of the cell type, proportional to the initial tissue contractility, and depends on RhoA-mediated myosin activity. Together, our results demonstrate that compressive stress can relax active tissue force, and points to a potential role of this feedback mechanism during morphogenetic events such as onco- or embryogenesis.

**Graphical abstract:**

**Figure:**
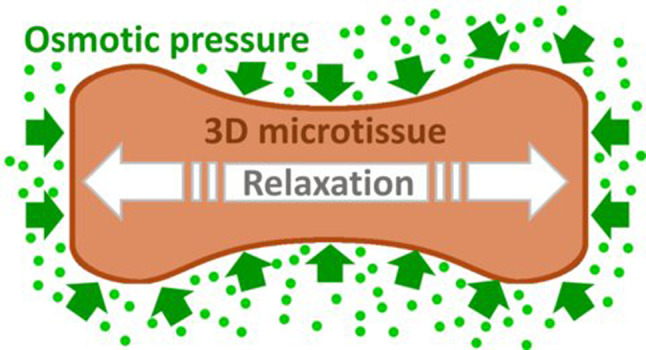
Schematic of osmotic pressure-induced relaxation of contractile microtissues

**Supplementary Information:**

The online version contains supplementary material available at 10.1140/epje/s10189-025-00497-0.

## Introduction

Tissue growth and function are driven by complex interactions between its constituent cells, extracellular matrix (ECM), and external stimuli [1]. Besides biochemical signals, cells receive and respond also to physical cues such as mechanical forces, fluid pressure, or the stiffness of the extracellular matrix [2, 3]. Cells respond to these external stimuli, e.g., by modulating their contractility, proliferation, metabolism or differentiation [1, 4, 5].

These mechano-biological feedback mechanisms are crucial for cancer progression [2, 6]. For example, cancer-associated fibroblasts (CAFs) in tumors secrete and remodel excessive amounts of extracellular matrix [7, 8], forming a rigid barrier that physically limits tumor growth [9, 10]. Moreover, the tumor is continuously contracted and compacted by active CAF forces [11, 12]. Both growth constriction and tumor compaction lead to the accumulation of solid stress pressure [9, 12, 13] which has been shown to limit or inhibit tumor growth [9, 13].

The pathophysiological roles of solid stress have been investigated in vitro by assessing the proliferation of tumor models in which solid stress, ranging from 0.5 to 20 kPa, was controlled by physical confinement [14, 15] or osmotic pressure [16–18]. These studies have demonstrated the crucial role of both the cytoskeletal machinery and the surrounding ECM in the response to compression or ECM reorganization [13, 19–22]. For instance, the regulation of proliferation by external pressure has been shown to depend on the tissue microstructure and mechanics, as growth is inhibited when compression pressure applies to both cells and ECM, whereas it remains unaffected when compression applies only to cells [20, 23].

As cells assess the mechanics of their environment by applying forces to it, the regulation of cell contractility by either proliferation-induced or CAF-generated solid stress may be key to understanding the feedback mechanism between solid stress and tumor growth. Indeed, the mechanical state of cells drives the ECM remodeling and stiffening [21, 22, 24], as well as cell proliferation [25–28], which in turn increases internal solid stress when it occurs in a confined environment. However the difficulty of assessing cell forces in 3D environments under compression leaves a notable gap in our understanding of its consequences on cell contractility.

While the active regulation of stress in microtissues under tension has been largely investigated [21, 29–32], in this work we focus on the regulation of contractile active stress in tissues under compression. We engineer 3D microtissues, i.e., microscale constructs of cells embedded in extracellular 3D collagen matrix, where the microstructure of the exogenous collagen is remodeled in fibers by the cells themselves [33–35]. The living material is suspended between two flexible cantilevers, whose deflection gives direct access to tissue contractility in real time [36–41]. The microtissues are then osmotically compressed using a culture medium supplemented with dextran, which is biologically inert and therefore exerts a persistent osmotic stress equivalent to an external mechanical compressive stress of identical magnitude [18].

To delineate the respective roles of cells and collagen in the mechanical response to pressure, we applied selective compression to both cells and extracellular matrix, or to individual components by modulating the molecular weight of dextran in the culture medium [23, 42]. Although an osmotic pressure leads to a compression stress, due to dehydration, we observe a counter-intuitive elongation of 3D tissues upon pressure. This compression-induced elongation only happens in 3D tissues composed of ECM and actively contracting cells, and only upon global compression, i.e., when both components are mechanically loaded. Furthermore, we demonstrate that this mechanical response of tissues to compression is cell type-independent but proportional to the initial tissue contractility. Together, these results highlight a unique approach to examine the effects of compression on 3D tissues and evidence the complex mechanical feedback loop between external pressure, ECM deformation and cell contractility.

## Results

### Regulation of tissue contractility upon compression

**Fig. 1:**
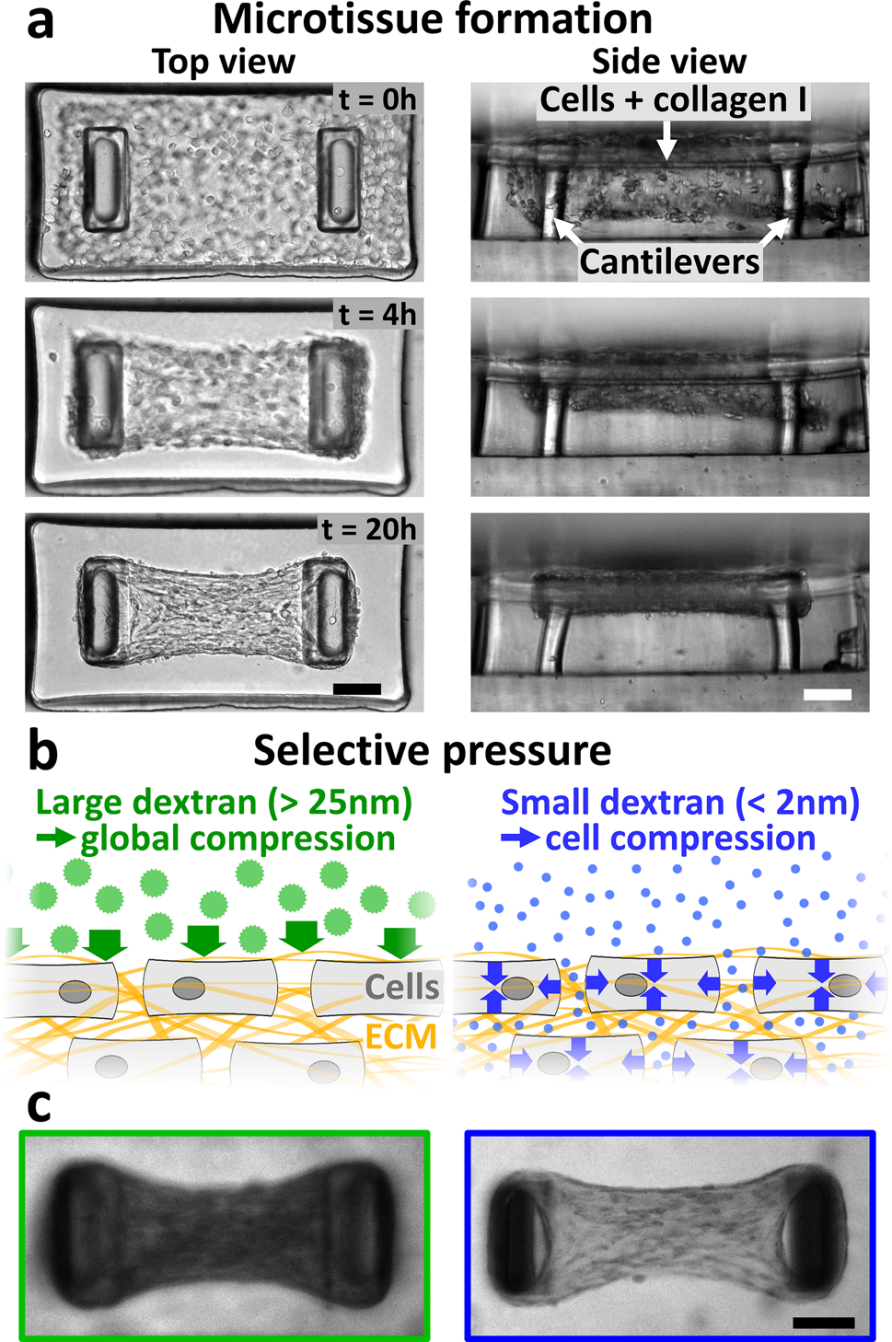
Microtissue engineering and selective compression. **a** Representative images of top view (left column) and side view (right column) illustrating the formation of a microtissue composed of NIH3T3 fibroblasts in collagen. Over time, the collagen matrix is spontaneously compacted by the fibroblasts to form a 3D microtissue suspended between T-shaped cantilevers. The deflection of the cantilevers, clearly visible after 20 h of formation, allows direct access to the tissue contractility. **b** Schematic of cells (gray) embedded in collagen (yellow fibers) submitted to selective compression. Large dextran osmolytes (green discs) do not permeate collagen and induce a global tissue compression (represented by green arrows), whereas small dextran osmolytes (blue dots) infiltrate collagen and induce a cell compression only (represented by blue arrows). **c** Confocal sections of representative microtissues with fluorescently labeled dextran molecules added to the culture medium. Large dextran molecules (gyration radius > 15 nm, left image) are excluded from the microtissue, which appears darker than its surroundings. By contrast, small dextran molecules (gyration radius < 2 nm, right image) permeate the microtissue, which appears nearly as bright as its surroundings. Scale bars are 100 μm

To engineer microtissues, we use arrays of 800 × 400x200 µm wells within a PDMS mold. We pipette a suspension of cells and monomeric neutralized collagen I in these microwells (Fig. 1a). We used either mouse colon adenocarcinoma CT26 cells to model tumor cells, NIH3T3 fibroblasts to model inactivated fibroblasts, which are the vast majority of CAF precursors [8], or human primary cancer-associated fibroblasts. Once polymerized, the collagen is compacted by the cells. Two T-shaped, flexible cantilevers incorporated within each microwell anchor the contracting collagen matrix, leading to the formation of a microtissue suspended between the top of the pair of cantilevers. The forces generated by the cells and acting on the surrounding collagen matrix result in a global tissue force that deflects the flexible cantilevers (Fig. 1a). Using linear bending theory and experimental measurements, we calibrated the spring constant of the cantilevers, which was then used to relate the measured cantilever deflections to the associated tissue-generated force [36–38].

Once the tissue is formed and the produced force is stabilized, we used dextran osmolytes to compress the microtissue. Dextran macromolecules are biologically inert polysaccharides that do not readily enter the cytoplasm. Depending on their molecular weight—and therefore their size—they may be unable or only partially able to diffuse into the interstitial space. Consequently, smaller dextran molecules remain mostly in the extracellular space, resulting in an extracellular–intracellular concentration gradient, whereas larger dextran molecules stay predominantly outside the tissue, resulting in an extra tissue-interstitial gradient [16, 23, 42, 43]. In both cases, these gradients induce a persistent osmotic stress [16, 44]. Previous studies have established that 2 MDa dextran molecules with a gyration radius larger than 25 nm [45] do not permeate the cell-compacted collagen and thus compress the whole tissue, whereas small, 10 kDa dextran molecules with a gyration radius below 2 nm [45] infiltrate the collagen network and compress the cells only (Fig. 1b–c) [23, 42].

**Fig. 2:**
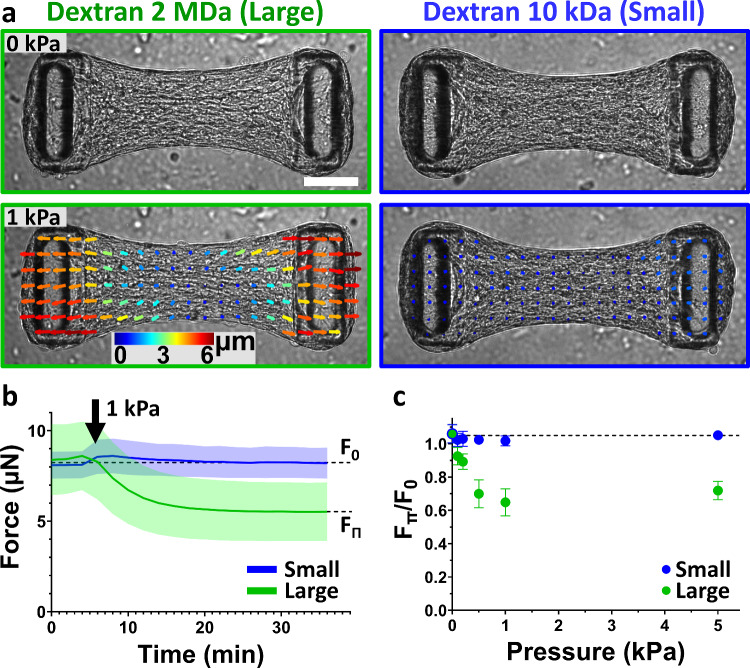
Microtissues uniaxially elongate under global but not cell compression. **a** 30 min after a 1 kPa global compression using large, 2 MDa dextran molecules (left column, outlined in green), a NIH3T3 microtissue relaxes, as shown by a representative PIV-tracking of the displacements, whereas a 1 kPa cell compression using small, 10 kDa dextran molecules has no effect (right column, outlined in blue). Scale bar is 100 µm. **b** Temporal evolution of the force generated by microtissues submitted to 1 kPa compression using large (green curve) or small (blue curve) dextran osmolytes. **c** Tissue relaxation, quantified by the force at equilibrium under pressure F_Π_ normalized by the initial force F_0_, i.e., F_Π_/F_0_, in function of the applied global (green) or cell (blue) compression. In control experiments (0 kPa), fresh culture medium without dextran was added to the microtissues. Data are the average of *n* > 25 microtissues over 2 independent experiments ± SD

When applying a tissue compression Π = 1 kPa to NIH 3T3 microtissues using large dextran, we observed a transient, transverse (i.e., perpendicular to the long axis of the tissue) compression followed by a tissue elongation of 3.5 ± 1.1% (12.7 ± 3.8 µm) (Fig. 2a and Supp. Movie 1). The force as measured by the pillar deflection decreased from its initial baseline level F_0_ = 8.4 ± 1.9 µN to F_Π_ = 5.5 ± 1.6 µN in approximately 15 min (Fig. 2b). This tissue elongation and force relaxation was reversible, as tissue force increased back to its initial level after dextran removal, but at a slower rate, in approximately 60 min (Supp. Figure 1).

This relaxation could either be an active response from the cells in the form of a reduced acto-myosin contractility, or the result of a passive elongation of the extracellular matrix within the tissue by the lateral osmotic compression. Confocal images of microtissues simultaneously stained for actin, collagen and DNA showed a compacted collagen core populated with fibroblasts, surrounded by a densely cellularized peripheral shell (Supp. Movie 2). Actin stress fibers, collagen fibers and nuclei were found predominantly aligned with the tissue contraction direction (i.e., along the axis between the two cantilevers, Supp. Movie 2), consistent with fibroblasts aligning and remodeling their extracellular matrix to align with the principal maximal strains developed during tissue formation [29, 33, 46, 47]. This anisotropic architecture was previously shown to correlate with anisotropic mechanical properties, as well as anisotropic contractility [32, 38]. Since microtissues elongate almost exclusively longitudinally, i.e., along their long axis between the two cantilevers, and cells are mainly aligned along the same axis, we propose the hypothesis that the osmotic pressure-induced elongation is not caused by passive volume-conserving effects stemming from the transient lateral tissue compression, but rather it is caused by a relaxation of the active, acto-myosin-generated forces as a result of tissue compaction.

We first tested whether cells were directly sensitive to osmotic pressure by applying a pressure Π = 1 kPa selectively on the cells only using small dextran. We also observed a transient compaction of the tissue in the transverse direction (perpendicular to the long axis of the tissue), but in contrast to the response to large dextran, this transient compaction was faster and was not followed by any tissue elongation or force relaxation (F_0_ = F_Π_ = 8.1 ± 0.7 µN) (Fig. 2a–b and Supp. Movie 3). This result is consistent with the notion that cells are not directly sensitive to osmotic compression, but rather they are sensitive to the mechanical compression of the tissue, which induces active relaxation of the cells when compressed.

To test this hypothesis further, we measured the dependence of tissue relaxation in response to different pressure levels. By varying dextran concentration, we applied a compressive stress ranging from 0.1 to 5.0 kPa to microtissues while monitoring tissue contractility. This stress range corresponds to pathophysiological tissue stress induced by hydrostatic pressure [48], confined tumor growth [13, 49] or active compression by CAFs surrounding cancer cells [9]. With large dextran, force relaxation became more pronounced with increasing pressure up to 1 kPa and then plateaued (Fig. 2c). By contrast, we did not detect any significant changes in force when applying cell compression with small dextran, regardless of pressure (Fig. 2c). Together, these results point again toward an active relaxation of cell contractility in response to compression, as passive tissue elongation by the action of increasing compressive stress would be expected to monotonically increase. Also, these results confirm that force relaxation is not caused by osmotic pressure applied directly to the cells but requires the pressure to act at the tissue level.

In order to delineate the respective roles of cell and matrix within the tissue, we next investigated the impact of pressure on isolated cells and decellularized matrix.

### Without each other, neither cells nor matrix relax under pressure

**Fig. 3:**
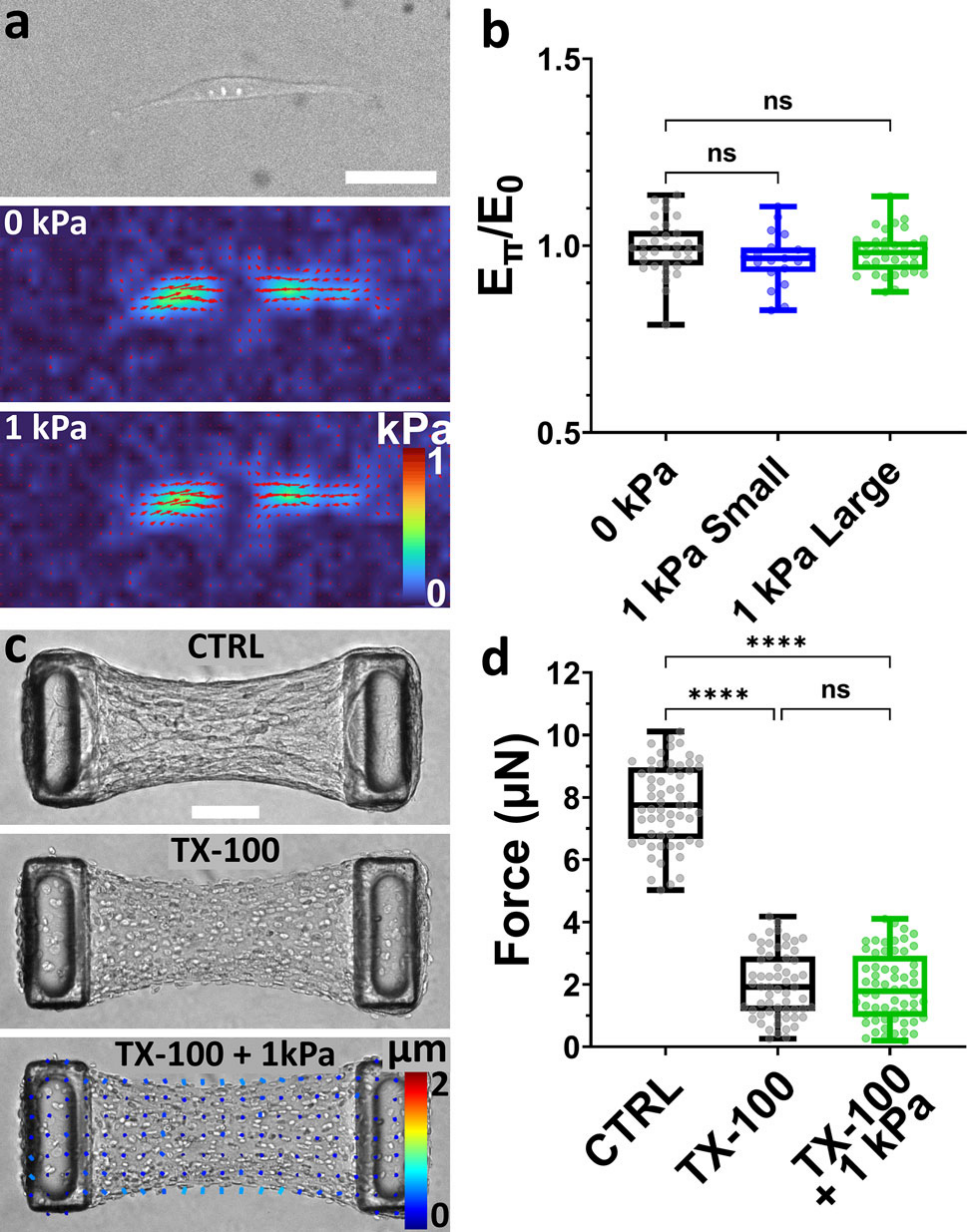
Single cells or decellularized matrix do not relax under osmotic pressure. **a** Representative brightfield image and corresponding stress maps of a NIH3T3 fibroblast spread on a soft PDMS substrate before and after application of a 1 kPa pressure using large, 2MDa dextran. Scale bar is 50 µm. **b** Contractile energy exerted by fibroblasts on the substrate under pressure (E_Π_) applied with either small (in blue) or large (in green) dextran, normalized by the initial contractile energy E_0_. In control experiments (0 kPa, in black), fresh culture medium without dextran was added to the cells. Data are presented as box plots superimposed with a dot plot of the data distribution with *n* > 19 cells over 2 independent experiments. **c** Representative brightfield images of a NIH3T3 microtissue (CTRL), decellularized using 0.5% Triton X-100 (TX-100), and 30 min after the application, once decellularized, of a 1 kPa pressure using large, 2 MDa dextran (TX-100 + 1 kPa). The bottom image is superimposed with a PIV-tracking of the displacements. Scale bar is 100 µm. **d** Corresponding tissue force. Data are presented as box plots superimposed with a dot plot of the data distribution with *n* = 40 microtissues over 2 independent experiments. n.s. stands for non-significant (i.e., *P* > 0.05). ****P < 0.0001 between conditions

To assess the contractile response of single cells to osmotic pressure, we quantified the forces generated by single fibroblasts spread on a soft PDMS substrate (Young’s modulus E = 15 kPa) grafted with 0.2 µm diameter fluorescent beads as fiducial markers and coated with fibronectin. Of note, 2D PDMS substrates were used instead of a 2D polyacrylamide substrate, a 3D PEG or a 3D collagen hydrogel as these hydrogels would be compressed by osmotic pressure, whereas PDMS is not affected. Cell forces and overall contractile elastic energy was calculated from substrate deformation, induced by cell-generated forces, determined from analysis of fluorescent bead images before and after cell removal [50–53]. We found that cell morphology, force localization and overall contractile energy E_C_ were unaffected by the application of a 1 kPa osmotic pressure using either small or large dextran (Fig. 3a–b), confirming that cells are not directly sensitive to osmotic pressure at that level.

To elucidate the contribution of the collagen matrix, we decellularized the microtissue using the surfactant Triton X-100. Such decellularization process was previously shown not to affect microtissue stiffness and collagen integrity [21, 54]. Contractility was drastically reduced upon decellularization, however the forces measured by pillar deflection did not fall to zero, likely because the collagen matrix was compacted and cross-linked during tissue formation and maturation [21, 38]. We then submitted decellularized microtissues to an osmotic pressure using large, 2MDa dextran. We observed a slight compression of the tissue indicating that cell-compacted collagen laden with dead cells is not permeable to large dextran molecules, and is therefore compressed by osmotic pressure. However, we did not measure any significant displacement of the pillar tips and hence no force changes (Fig. 3c–d and Supp. Movie 4), confirming that the elongation of intact microtissues is not driven by a passive mechanical response of the collagen under compression. This interpretation is further supported by our observation that the collagen density did not influence the relaxation of microtissues submitted to osmotic pressure (Supp. Figure 2).

Overall, these results demonstrate that neither the cells alone nor the matrix alone relax under weak osmotic pressure. We therefore hypothesize that the osmotic pressure-induced elongation of microtissues is caused by the relaxation of active cell forces, in response to a mechanical compression of their surroundings.

### Pressure-induced relaxation depends on cell contractility

**Fig. 4:**
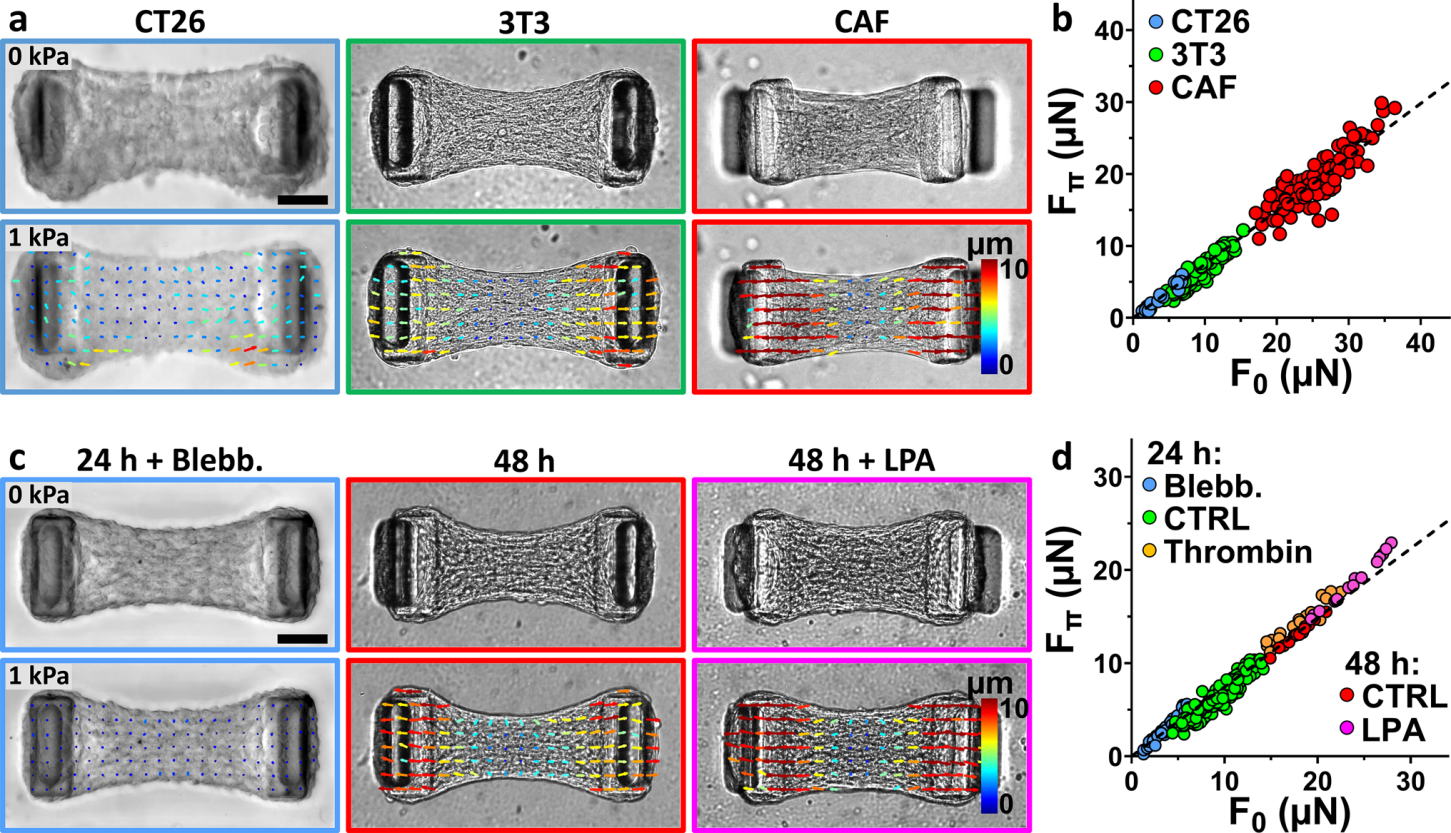
Pressure-induced relaxation correlates with the initial tissue force **a** Representative brightfield images of microtissues composed of murine colon carcinoma CT26 cells (blue frame), murine NIH3T3 fibroblasts (green frame) or human primary cancer-associated fibroblasts (CAF, red frame) before (top row) and after (bottom row) the application of a 1 kPa pressure using large, 2 MDa dextran. The bottom image is superimposed with a PIV-tracking of the displacements. **b** Corresponding scatter plot of the pressured (F_Π_) versus the initial (F_0_) tissue force for the three cell types, superimposed with a linear regression of the data (slope = 0.74, *R*^2^ = 0.97, Pearson coefficient *r* = 0.98). **c** Representative brightfield images of 3T3 microtissues after 24 h of formation and incubated for 1 h with 10 µM of blebbistatin (blue frame), after 48 h of formation (red frame) or after 48 h of formation and incubated for 1 h with 10 µM of lysophosphatidic acid (48 h + LPA, magenta frame) before (top row) and after (bottom row) the application of a 1 kPa pressure using large, 2 MDa dextran. The bottom image is superimposed with a PIV-tracking of the displacements. **d** Scatter plot of the pressured (F_Π_) versus the initial (F_0_) tissue force for 3T3 microtissues either after 24 h of formation and incubated for 1 h with 10 µM of blebbistatin (Blebb., blue dots), growth medium (CTRL, green dots) or 1 U/mL of thrombin (Thrombin, orange dots); or after 48 h of formation and incubated with growth medium (CTRL, red dots) or 10 µM of lysophosphatidic acid (LPA, magenta dots). The dot plot is superimposed with a linear regression of the data (slope = 0.74, *R*^2^ = 0.97, Pearson coefficient *r* = 0.99). Scale bars are 100 µm

To probe the response of different tissues on pressure-induced relaxation, we applied a 1 kPa osmotic pressure on microtissues composed of different cell types known to exert different levels of contractility: murine colon carcinoma CT26 cells, murine NIH3T3 fibroblasts and human primary cancer-associated fibroblasts (CAFs) (Fig. 4a). After 24 h of formation, CT26 microtissues reached an initial force F_0_ of 3.4 ± 2.0 µN while 3T3 microtissues generated 9.3 ± 2.4 µN (Fig. 4b). Because primary human CAFs present a myofibroblastic phenotype [55, 56], they are larger and considerably more contractile than CT26 or 3T3 cells [57]. Consequently, we halved the cell density in CAF-based microtissues, to prevent the tissue from tearing or slipping off the cantilever. They still produced an initial force F_0_ of 25.4 ± 4.5 µN after 24 h of formation (Fig. 4b).

Upon osmotic pressure application, all three types of microtissues relaxed, suggesting that pressure-induced relaxation is not tissue- or cell-type specific. However, the amplitude of the force relaxation linearly increased with the contractile force: weakly contractile CT26 microtissues relaxed by 1.0 ± 0.5 µN, 3T3 microtissues by 3.0 ± 0.8 µN and highly contractile CAF microtissues by 6.2 ± 2.3 µN, with a cell type-independent 26% relaxation of the initial force under pressure (Fig. 4b and Supp. Figure 3). Put differently, the final force after pressure application (F_Π_) reached 74% of the initial force (F_0_), in all tissues and regardless of cell type: F_Π_ = 0.74 F_0_.

We next tested if this relationship between initial and final force also holds within a single tissue when acto-myosin-generated forces are pharmacologically altered. We cultured 3T3 microtissues for 24 h and 48 h, as maturation duration has been shown to strongly increase the remodeling and cross-linking of collagen by the cells, impacting overall tissue mechanics and especially contractility [29, 33, 38]. We incubated the tissues for 1 h with either growth medium (control condition), blebbistatin (a myosin ATPase inhibitor that lowers cell contractility [36, 58, 59]), lysophosphatidic acid (LPA, a phospholipid stimulant of myosin activity via RhoA activation, which increases cell contractility [60–63]) or thrombin (an enzyme increasing RhoA-mediated myosin-generated contraction [62, 64–67]) (Fig. 4c and Supp. Figure 4). Depending on treatment, we obtained 3T3 microtissues with initial forces F_0_ ranging from 3.5 ± 1.2 µN (24 h of maturation + 10 µM Blebbistatin) to 23.6 ± 2.8 µN (48 h of maturation + 10 µM of LPA) (Supp. Figure 3). When subjected to an osmotic pressure of 1 kPa, all microtissues relaxed on average by 26%, i.e., the amplitude of relaxation (i.e., F_0_—F_Π_) was directly proportional to the initial force F_0_ (Fig. 4c–d, Supp. Figure 3 and Supp. Figure 4), thus confirming that the pressure-induced force relaxation is regulated by myosin activity. Of note, the spring constant of cantilevers has been shown to influence tissue force and collagen compaction [29, 36, 38, 39, 68]. We therefore checked whether it had any impact on the pressure-induced relaxation, but we still found the same 26% relaxation (Supp. Figure 5).

## Discussion

Cells constantly generate forces to migrate, to communicate mutual positions, to rearrange the surrounding ECM and neighboring cells, while also reacting to their environment, resulting in a complex feedback loop [69–71]. This interplay between internal and external forces is especially crucial for tumor progression [2, 6, 9, 13]. However, the impact of cell proliferation-induced compression on cell contractility, which in turn regulates cell proliferation, remains elusive.

Here we applied osmotic compression to 3D microtissues composed of cells and collagen I while simultaneously measuring microtissue shape and contractility. In usual tensional probes, cells rapidly reinforce their cytoskeleton and increase the forces they generate in response to increased external forces [1, 5, 72, 73]. Conversely, we observed a fast and significant relaxation of microtissues subjected to a global osmotic compression and the amplitude of relaxation was correlated to the level of the imposed osmotic pressure.

We first tested whether this relaxation was an active, cell-driven mechanism, or a passive, collagen-driven one. We applied selective compression on cells only using small dextran molecules, capable of permeating between the cells and through the collagen network, but we did not measure any relaxation. These results suggest that the collagen matrix acts as a pressure sensor for cells, in agreement with our previous work showing that global compressions regulate spheroid growth and cell motility, whereas selective compression of cells only has no effect [23].

In the literature it is reported that large osmotic and mechanical pressures (of the order of hundreds of kPa) are needed to induce cell and nucleus deformation [74, 75] and interfere with cell proliferation [76–79]. The osmotic pressures we applied, ranging from 0.1 to 5 kPa, were proved to be too weak to induce measurable single cell or nucleus deformation [42]. However, cell-compacted collagen, whose elastic modulus is of the order of a few tens of kPa [21, 24, 38, 80], could be deformed by osmotic pressures in the kPa range, i.e., close to solid stress existing in tumors tissues [12, 13].

By quantifying the forces generated by single cells spread on 2D PDMS substrates, we confirmed that cell shape and contractility were not affected by osmotic pressure in the weak compression regime we adopt. To investigate the mechanical behavior of the collagen component only, we decellularized already assembled microtissues to obtain passive collagen constructs containing dead cells. We thus observed a lack of relaxation under osmotic pressure, confirming that collagen is not directly elongating under pressure.

If cells respond to a small deformation of collagen by decreasing their contractility, we hypothesized that this response should depend on the initial contractility of the tissue, as the mechanical properties of collagen are highly dependent on its stress state [24, 38, 69, 80]. Using three different cell types as well as different drugs affecting myosin activity, we demonstrated that pressure-induced relaxation is intrinsic to cell/collagen-based constructs and is proportional to the tissue’s initial contractility, i.e., a force under a pressure of 1 kPa is always around 26% lower than the initial force. We speculate that collagen compression may lead to changes in ligand density, pore size or network stiffness [24, 69] that have been shown to affect cell forces [69, 81, 82].

Overall, our results evidence a general, robust and rapid mechanosensitive response of tissues to osmotic pressure, driven by myosin activity. This mechanism could be similar to the stretch-induced fluidization of 2D cell cultures [83, 84], 3D cell/collagen-based microtissues [31] or ex vivo tissue strips [85], where external stretch leads to actin depolymerization, resulting in cell or tissue softening accompanied by a decrease in contractility [31, 83, 85]. An alternative hypothesis would be that osmotic pressure may lead to buckling of collagen or actin fibers [74, 86, 87], which was previously shown to induce drastic softening of collagen [69, 88] or severing and depolymerization of actin [86, 89], respectively.

This mechanosensitive response potentially underlies the growth inhibition previously demonstrated in tumors under compression [9, 14, 19, 20, 23], as cell contractility has been shown to regulate cell proliferation [25–28]. Thus, a thorough characterization of the mechanics of cell-compacted collagen networks under compression could be key to furthering our understanding of the relationship between compression, tissue mechanics and cell contractility. Along similar lines, experimental developments aimed at combining tissue force measurement with other approaches to pressure application, such as hydrostatic pressure [48, 90, 91] or physical confinement [15, 16], would be potentially relevant in helping to better understand the regulation of cell contractility by solid stress. Such characterization could therefore put into perspective our current knowledge of volume change and stress distribution in tumor spheroids under self- or externally generated pressure [9, 23].

In conclusion, as cell shape and contractility are key regulators of cell proliferation, epithelial-mesenchymal transition and differentiation [6, 25, 92–94], our approach combining microtissue engineering, real-time tissue force monitoring and selective osmotic pressure could pave the way to test whether the active decrease in cell contractility under pressure is a key mechanism during onco- and morphogenesis.

## Materials and methods

### Cell culture and reagents

Mouse colon adenocarcinoma CT26 cells (ATCC CRL-2638) and NIH 3T3 fibroblasts (ATCC CRL-1658) were cultured (< 15 passages) in culture medium composed of Dulbecco’s modified Eagle’s medium (DMEM, Gibco Invitrogen) supplemented with 10% (v/v) fetal bovine serum (FBS, Gibco Invitrogen), 100 U/ml of penicillin and 100 µg/ml of streptomycin (Gibco Invitrogen). Human primary cancer-associated fibroblasts (CAF) were kindly provided by D. Matic Vignjevic (I. Curie, Paris). They were isolated from an upper rectum lieberkuhnian adenocarcinoma and immortalized as described earlier [9] at Institut Curie Hospital, Paris, with the patient’s written consent and approval of the local ethics committee. CAFs were cultured in the same culture medium supplemented with 1% (v/v) Insulin–Transferrin–Selenium (ITS, Sigma). All cell types were kept at 37 °C in an atmosphere saturated in humidity and containing 5% CO_2_.

Triton X-100 (Sigma), blebbistatin (Sigma), lysophosphatidic acid (Sigma) and thrombin (ThermoFisher Scientific) were introduced in the culture medium 1 h prior to the application of osmotic pressure at a 0.5% (v/v), 10 μM, 10 µM and 1U/mL concentration, respectively.

### Device fabrication, calibration and microtissue engineering

The microtissues were engineered within polydimethylsiloxane (PDMS, Sylgard 184, Dow Corning) microwells 800 µm long, 400 µm wide and 200 µm deep. Each microwell contains two T-shape cantilevers that constrain self-assembly and ensure good anchorage of the microtissue. Microwells were made by PDMS replication of SU-8-based masters microfabricated as described previously [36–38]. Briefly, successive layers of negative and positive photoresist (Microchem) were spin coated, insolated and baked to create multilayers templates. PDMS microwells were then molded from the SU-8-based masters by double replication after silanization with trichloro(1H,1H,2H,2H-perfluorooctyl)silane (Sigma) to facilitate subsequent release of PDMS from the template. PDMS stiffness was assessed through uniaxial tensile tests with an Instron 5848 Microtester (Instron). Cantilever spring constant *k* was calibrated with a capacitive MEMS force sensor mounted on a micromanipulator as described previously [33, 36, 38, 95] and found to be *k* = 0.45 ± 0.10 N/m.

PDMS microwells were sterilized in 70% ethanol and treated with 0.2% (m/v) Pluronic F127 (Sigma) for 2 min to reduce cell adhesion. A cooled suspension of 300,000 cells (CAF) or 600,000 cells (CT26 and 3T3) per mL of liquid neutralized collagen I from rat tail (Advanced Biomatrix) was then added to the microwells on ice and centrifuged to drive cells into the recessed wells. The collagen concentration was set at 2.0 mg/mL for all experiments except for Supp. Figure 2 where it was set at 1.5 or 2.5 mg/mL. Excess cell/collagen mixture was removed and the remaining constructs were polymerized at 37 °C for 9 min before the addition of culture medium. Microtissues were kept in the incubator for 24 h or 48 h prior to experiments. Over time, cells spread and compact the collagen matrix to form a microtissue suspended between the top of the pair of cantilevers.

### Osmotic pressure

Osmotic pressure is exerted by adding to the culture medium a well-defined amount of dextran (Sigma), a biologically inert polysaccharide. As such, its concentration gradient cannot be balanced by the activity of ion pumps, channels or endocytosis and it therefore exerts a persistent osmotic stress [16, 23, 42, 43]. We used large dextran (MW = 2 MDa, hydrodynamic radius > 25 nm [45]) which cannot penetrate the cell/collagen network to apply osmotic stress to the entire microtissue (Fig. 1b left schematic). We used small dextran (MW = 10 kDa, hydrodynamic radius < 2nm [45]) that can diffuse between cells and through collagen to apply pressure only to cells (Fig. 1b right schematic). The relationships between dextran size, concentration and resulting osmotic pressure are given in Table 1.

**Table 1:** Range of osmotic pressure used and corresponding concentrations of large, 2 MDa dextran and small, 10 kDa dextran. For small, 10 kDa dextran, the osmotic pressure is directly derived from the van’t Hoff formula Π = *c*.*R*.*T*, with *c* the molar concentration of dextran, *R* the ideal gas constant, and *T* the absolute temperature. Because of their size, 2 MDa dextran molecules are self-interacting and do not obey the van’t Hoff equation. The relationship between concentration and pressure has been previously calibrated [42, 96–98]

Osmotic pressure (kPa)	[2 Mda Dextran] (g/L)	[10 kda Dextran] (g/L)
0.1	3.2	0.4
0.2	5.9	0.8
0.5	12.4	2.0
1.0	20.6	4.0
5.0	54.8	20.0

### Microscopy and image analysis

Brightfield imaging was performed using either a Nikon Eclipse TI-2 inverted microscope equipped with an Orca flash 4.0 LT CMOS digital camera (Hamamatsu), a CFI S Plan Fluor ELWD 20x/0.45 objective (Nikon) and controlled with NIS Elements software (Nikon), or a Nikon Ti-E inverted microscope equipped with a Neo sCMOS camera (Andor), a Plan Fluor10x/0.30 objective (Nikon) and controlled with IQ3.1 software (Andor). Both microscopes are equipped with an incubator that maintains an atmosphere of 37 °C, saturated with humidity and containing 5% CO_2_.

The force generated by individual microtissues before, during and after osmotic pressure was assessed from the deflection of the cantilevers, as previously described [36–38]. Briefly, the deflection *d* was determined by comparing the position of the cantilevers’ T-shaped caps with their initial position (i.e., before tissue formation). Brightfield images were taken every 2 min and the position of the top of the cantilevers was tracked using a previously developed MATLAB script [38]. Tracking results were checked visually and faulty tracking was either redone by hand or discarded. The force *F* generated by the microtissue was deduced from the average deflection *d*_*AVG*_ of the two cantilevers and spring constant *k* as follow: *F* = *k*.*d*_*AVG*_. Only tissues that were uniformly anchored to the tips of the two cantilevers throughout the duration of the experiments were included in the analysis.

Displacement fields were calculated from the brightfield images using a MATLAB particle image velocimetry (PIV) toolbox (https://pivlab.blogspot.com/) [99], as previously described [38]. Briefly, the algorithm cross-correlates small interrogation areas of a pair of images (reference image at *t* = 0 and image of interest) in the frequency domain using FFT to determine the most likely displacement vector (u,v) of the particle at position (x,y) in the interrogation area. The 1500 × 450 pixels (660 × 330 µm) images were analyzed in four successive passes with decreasing interrogation areas, from 128 × 128 to 32 × 32 pixels leading to a final resolution of 14 µm. Missing vectors were replaced by interpolated data [100], outliers were filtered using a local normalized median filter [101], and the noise was reduced using a penalized least squares method [102]. It should be noted that cell migration during the duration of a pressure-induced relaxation was considered negligible compared to cell deformation. For readability reasons, only half of the vectors are represented in the displacement-superimposed images.

### Traction force microscopy (TFM) of single cells

Force measurements were performed using a method described previously [103]. In short, fluorescent beads were grafted to the surface of a soft PDMS substrate with 15 kPa rigidity and images of those beads were taken before and during the application of osmotic pressure. Soft PDMS (Dowsyl CY 52-276) substrates were prepared by mixing CyA and CyB components at 1:1 ratio before spin-coating 0.1 g for 30s at 500 rpm on a 35 mm glass bottom Fluorodish (World Precision Instruments) to achieve a flat 60—100 μm flat layer. After curing at 80 °C for 2 h, the substrate was silanized with 10% (3-Aminopropyl) trimethoxysilane (APTES, Sigma) in ethanol for 15 min before grafting carboxylated 200 nm fluorescent beads (647 nm, Invitrogen) at a 4:500 dilution in deionized water. Bovine fibronectin (50 μg/ml, Sigma) was incubated on the substrate for 1 h prior to cell seeding. Between each step, the samples were rinsed 3 times with PBS. Approximately 50,000 cells were seeded and let to adhere overnight. At the end of the experiment, cells were removed using a deionized water-based osmotic shock and an unstressed reference image of the beads was taken.

The displacement field analysis was obtained using a homemade algorithm based on optical flow [104, 105]. Cellular traction forces were calculated using Fourier transform traction cytometry with zero-order regularization [104, 106, 107] under the assumption that the substrate is a linear elastic half-space and considering only displacement and stress tangential to the substrate. To calculate the strain energy stored in the substrate, the scalar product of the stress and displacement vector fields was integrated over the surface of the whole cell. The algorithm was implemented in Python and is available in [104, 105].

TFM movies were recorded on a Nikon Ti2 eclipse microscope equipped with a Yokogawa CSU-W1 spinning disk confocal scanner and a Teledyne Photometrics Prime BSI camera at 20 × magnification (NA = 0.75).

### Statistics

For each box plots, the box extends from the 25th to 75th percentiles, the median is plotted as a line inside the box, the whiskers extend to the most extreme data point and the data distribution is superimposed as a dot plot. Statistical significances were determined by one-way analysis of variance (ANOVA) corrected for multiple comparisons using Tukey test with Prism (GraphPad).

## Supplementary Information

Below is the link to the electronic supplementary material.

Supplementary file1: Supp. Movie 1. 3T3 microtissue response to tissue compression. Time lapse of a representative 3T3 microtissue submitted to a 1 kPa osmotic pressure using large, 2MDa dextran molecules at t = 12 min. Upon osmotic pressure, the PIV-tracking of the displacements highlights the transient, transverse compression of the tissue, followed by its elongation. (AVI 5658 KB)

Supplementary file2: Supp. Movie 2. Confocal reconstruction of an 3T3 microtissue. Merged Z-stack (top) of a representative, unpressured 3T3 microtissue stained for actin (in green), collagen (in magenta) and nuclei (in blue), highlighting the anisotropic orientation of actin and collagen fibers along the x-axis. The right column shows magnifications of the central region for each staining. Scale bars are 50 µm. (AVI 2321 KB)

Supplementary file3: Supp. Movie 3. 3T3 microtissue response to cell-only compression. Time lapse of a representative 3T3 microtissue submitted to a 1 kPa osmotic pressure using small, 10kDa dextran at t = 12 min. Upon osmotic pressure, the PIV-tracking of the displacements highlights the transient compaction of the tissue in the transverse direction, not followed by any tissue elongation. (AVI 5447 KB)

Supplementary file4: Supp. Movie 4. Decellularized 3T3 microtissue response to tissue compression. Time lapse of a representative 3T3 microtissue decellularized with 0.5 % Triton X-100 and submitted to a 1 kPa osmotic pressure using large, 2MDa dextran molecules at t = 12 min. Upon osmotic pressure, the PIV-tracking of the displacements highlights the slight compression of the tissue, not followed by any tissue elongation. (AVI 4616 KB)

Supplementary file5 (DOCX 1176 KB)

## Data Availability

Because of the large file size, the datasets generated and/or analyzed during the current study are available from the corresponding author on request. A response will be provided in less than 2 weeks.
