# Supplementary material for: Osmotic pressure induces unexpected relaxation of contractile 3D microtissue
Source: Eur Phys J E Soft Matter. 2025 Jun 24;48(6-7):34. doi: 10.1140/epje/s10189-025-00497-0 (PMC12187822; doi:10.1140/epje/s10189-025-00497-0)
Supplement: Supplementary file 5 — Supplementary file5 (DOCX 1176 KB) [file 10189_2025_497_MOESM5_ESM.docx]

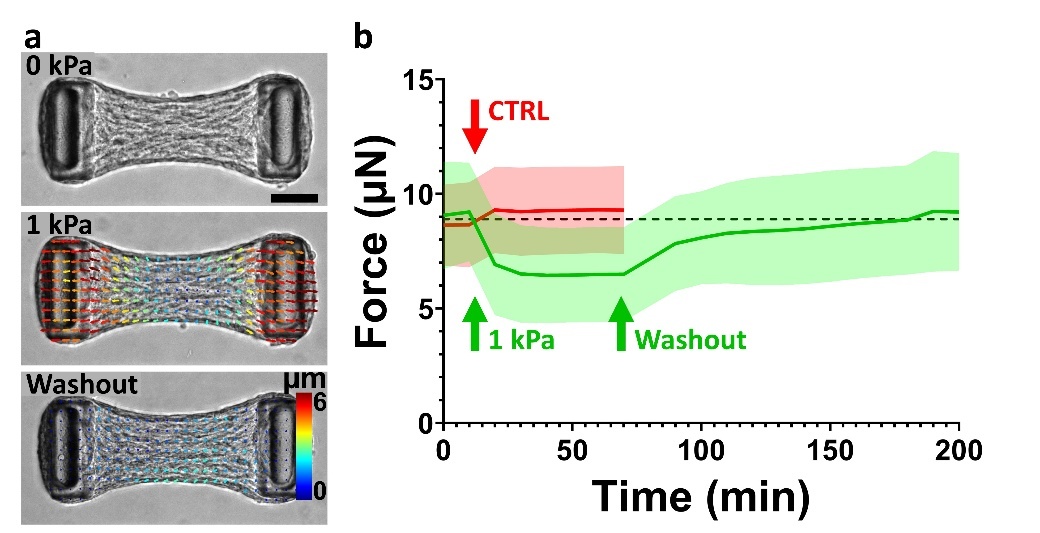


**Supp. Fig. *1*. Pressure-induced relaxation is reversible.** (a) PIV-tracking of the relaxation and recovery of a representative NIH3T3 microtissue upon the application and removal of a 1 kPa global compression using large, 2 MDa dextran molecules, respectively. (b) Temporal evolution of the force generated by microtissues submitted either to the application of growth medium only (CTRL, in red), or to the application and washout of a 1 kPa compression using large dextran osmolytes (in green). Data are the average of n > 40 microtissues over 3 independent experiments ± SD. Scale bar is 100 µm.


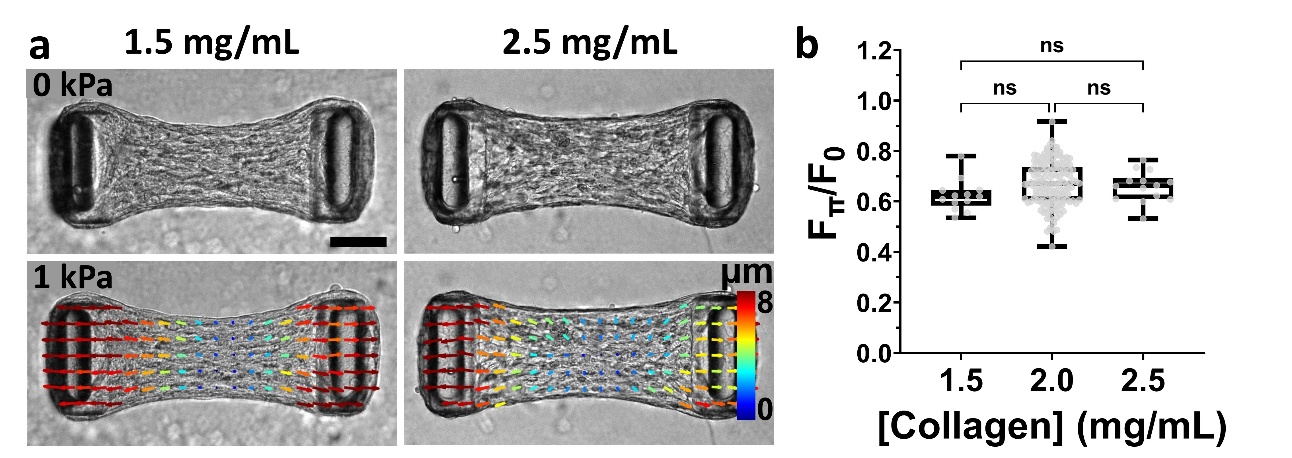


**Supp. Fig. *2*. Pressure-induced relaxation is independent of initial collagen density.** (a) PIV-tracking of the relaxation of representative NIH3T3 microtissues composed of 1.5 mg/mL (left column) or 2.5 mg/mL (right column) collagen upon the application of a 1 kPa global compression using large, 2 MDa dextran. (b) Tissue relaxation, quantified by the force under pressure F_Π_ normalized by the initial force F_0_, i.e. F/F_0_, as a function of the collagen concentration. Data are presented as box plots superimposed with a dot plot of the data distribution with n > 13 microtissues over 2 independent experiments. n.s. stands for non-significant (i.e. P > 0.05). Scale bar is 100 µm.


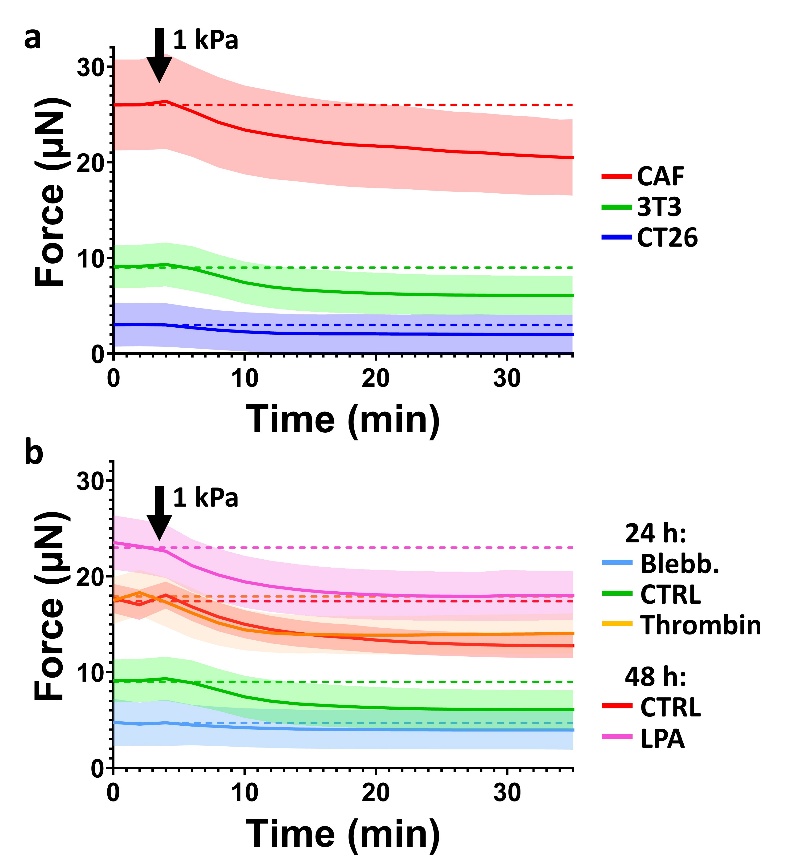


**Supp. Fig. *3*. Pressure-induced relaxation correlates with the initial tissue force** (a) Temporal evolution of the force generated by microtissues composed of murine colon carcinoma CT26 cells (blue line), murine NIH3T3 fibroblasts (green line) or human primary cancer-associated fibroblasts (CAF, red line) submitted to a 1 kPa pressure using large, 2 MDa dextran at t = 5 min. Data correspond to the dot plot presented in Fig. 4.b and are the average of n > 20 microtissues over > 2 independent experiments ± SD. (b) Temporal evolution of the force generated by 3T3 microtissues either after 24 h of formation and incubated for 1 h with 10 µM of blebbistatin (Blebb., blue line), growth medium (CTRL, green line) or 1 U/mL of thrombin (Thrombin, orange line); or after 48 h of formation and incubated with growth medium (CTRL, red line) or 10 µM of lysophosphatidic acid (LPA, magenta line); and submitted to a 1 kPa pressure using large, 2 MDa dextran at t = 5 min. Data correspond to the dot plot presented in Fig. 4.d and are the average of n > 15 microtissues over > 2 independent experiments ± SD.


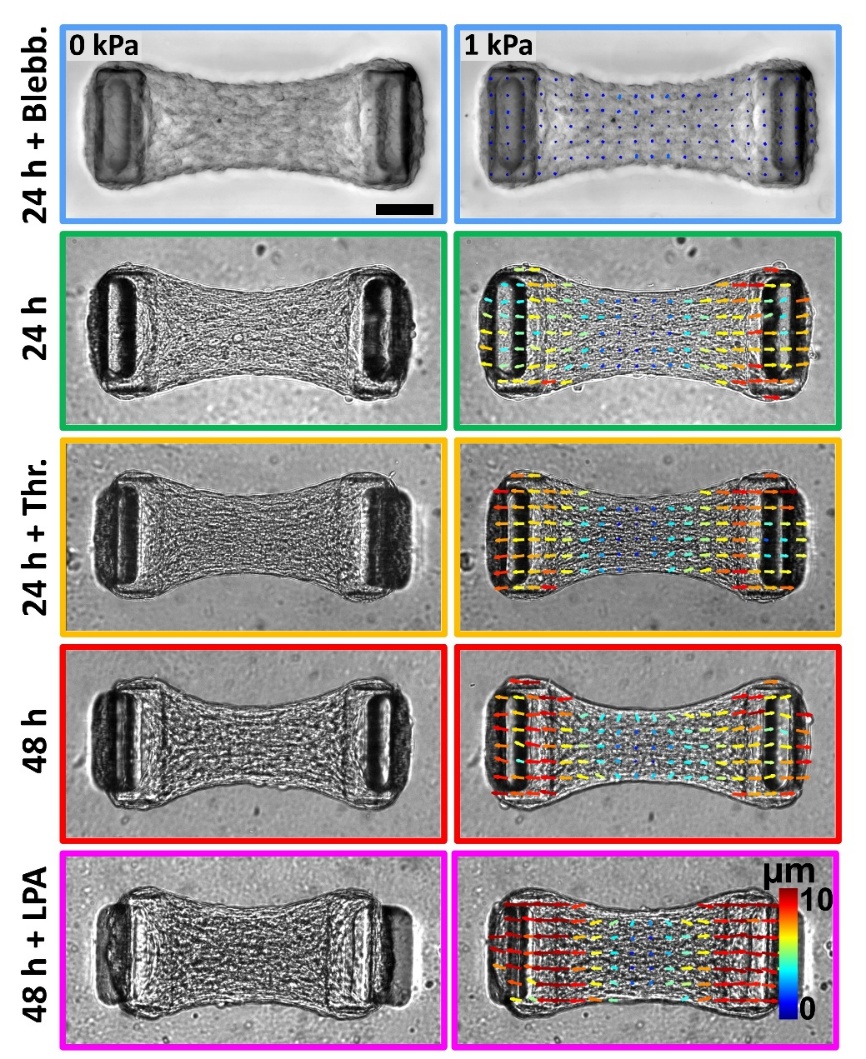


**Supp. Fig. *4*. Pressure-induced relaxation correlates with the initial tissue force** (a) Representative brightfield images of 3T3 microtissues after 24 h of formation and incubated for 1 h with either 10 µM of blebbistatin (24h + Blebb., blue frame), growth medium (24 h, green frame) or 1 U/mL of thrombin (24 h + Thr.), after 48 h of formation (48 h, red frame) or after 48 h of formation and incubated for 1 h with 10 µM of lysophosphatidic acid (48 h + LPA, magenta frame) before (left column) and after (right column) the application of a 1 kPa pressure using large, 2 MDa dextran. The image in the right-hand column is superimposed with a PIV-tracking of the displacements. Relaxation quantification is shown in Fig. 4. Scale bar is 100 µm.


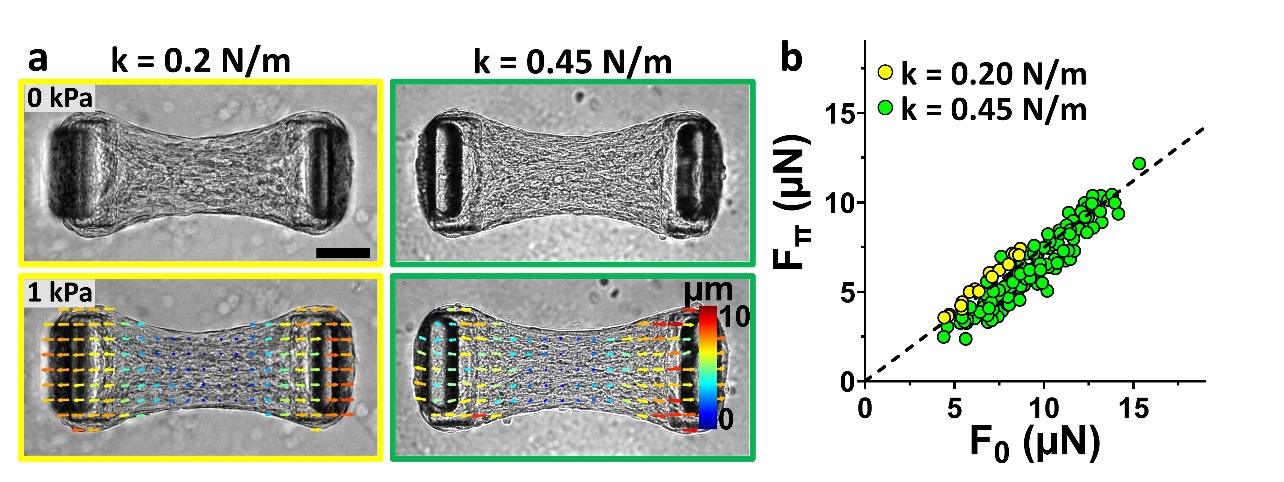


**Supp. Fig. *5*. Pressure-induced relaxation correlates with the initial tissue force** (a) PIV-tracking of the relaxation of representative NIH3T3 microtissues tethered to flexible (k = 0.20 N/m, yellow frame) or rigid (k = 0.45 N/m, green frame) cantilevers upon the application of a 1 kPa global compression using large, 2 MDa dextran. (b) Corresponding scatter plot of the pressured (F_Π_) versus the initial (F_0_) tissue force for the two conditions, superimposed with the linear regression of the data (presented in Fig. 4.d. Scale bar is 100 µm.
